# Session interest model for CTR prediction based on self-attention mechanism

**DOI:** 10.1038/s41598-021-03871-y

**Published:** 2022-01-07

**Authors:** Qianqian Wang, Fang’ai Liu, Xiaohui Zhao, Qiaoqiao Tan

**Affiliations:** 1 Shandong Women’s University, Jinan, China; 2 Shandong Normal University, Jinan, China; 3 Shandong Provincial Key Laboratory of Network Based Intelligent Computing, Jinan, China

**Keywords:** Electrical and electronic engineering, Computer science, Information technology, Scientific data

## Abstract

Click-through rate prediction, which aims to predict the probability of the user clicking on an item, is critical to online advertising. How to capture the user evolving interests from the user behavior sequence is an important issue in CTR prediction. However, most existing models ignore the factor that the sequence is composed of sessions, and user behavior can be divided into different sessions according to the occurring time. The user behaviors are highly correlated in each session and are not relevant across sessions. We propose an effective model for CTR prediction, named Session Interest Model via Self-Attention (SISA). First, we divide the user sequential behavior into session layer. A self-attention mechanism with bias coding is used to model each session. Since different session interest may be related to each other or follow a sequential pattern, next, we utilize gated recurrent unit (GRU) to capture the interaction and evolution of user different historical session interests in session interest extractor module. Then, we use the local activation and GRU to aggregate their target ad to form the final representation of the behavior sequence in session interest interacting module. Experimental results show that the SISA model performs better than other models.

## Introduction

It is a critical problem to predict the probabilities of users clicking on ads or items for many applications such as online advertising or recommender systems^1,2^. Cost per click (CPC)^3^ model is often used in advertising system. The accuracy of click-through rate (CTR) can influence the final revenue in CPC model. At the same time, displaying suitable advertisement to user can enhance their experience. Therefore, both academia and industry are concerned about how to design the CTR prediction models.

Modeling feature interaction is very critical in CTR prediction tasks. Recently, some effective methods ignore to capture user interest. User interest has an important influence on CTR prediction. In the fields with rich internet-scale user behavior data, such as online advertising, user’s sequential behaviors reflect user evolving interests. However, some models based on user interest overlook the intrinsic structure of the sequences. Multiple sessions make up a sequence. A session is a list of user behaviors that occur within a given time frame. The user behavior in each session is highly homogeneous, and the user behavior in different sessions is heterogeneous. Grbovic et al.^4^ found the session division principle that there is a time interval of more than 30 min. For example, the user mainly browses the T-shirt in the first half an hour as session 1, and browses the sneakers in the second half an hour as session 2. User shows different interests in session 1 and session 2.We can know the fact that people has a clear and unique intent at a session, but the interest usually changes when user start a new session.

Through the above observation, we propose Session Interest Model via Self-Attention (SISA) for CTR prediction, which uses multiple historical sessions to simulate the user’s sequential behavior in the CTR prediction task. At session division module, we naturally divide the user sequential behavior into sessions. At session interest extractor module, a self-attention mechanism with bias coding is applied to model each session. Self-attention mechanism gets the internal relationship of each session behavior, then, extracts the user interest in each session. Since different session interest may be related to each other or follow a sequential pattern, we use Gated Recurrent Unit (GRU) to capture interaction and evolution of user different historical session interests at session interacting module. Because different session interests have different effects on the target item, we utilize attention mechanism to achieve local activation and use GRU to aggregate their target ad to get the final representation of the behavior sequence.

The main contributions of this paper are as follows:

1. The user behavior in each session is highly homogeneous, and the behavior of user in different sessions is heterogeneous. We focus on user multiple session interest and propose a novel session interest model via self-attention (SISA). We can get more expressions of interest and more accurate prediction results.
2. We specially divide the user sequential behavior into sessions and design session interest extractor module. We employ a self-attention network with bias coding to obtain an accurate expression of interest for each session. Then we use GRU to capture the interaction of different session interests. At the same time, we exploit GRU with attentional update gate (AUGRU) to aggregate their target ad to find the influences of different session interests at session interacting module.
3. The experimental results show that our proposed model has great improvements over other models. At same time, the influence of key parameters and different variants is also explored, which proves the validity of the SISA model.

The rest of the paper is organized as follows. We discuss the related work in “Related work» and introduce the detailed architecture of proposed SISA model in “Material and methods”. Section “Experiments” verify the prediction effectiveness of the proposed model through experiments, and analyse the results. Furthermore, in “Conclusion”, we summarize the model presented in this paper and introduce the direction of future work.

## Related work

The CTR prediction problem is normal formulated as a binary classification problem. Logistic regression (LR)^5^ is a linear model that is used in the industry. Kumar et al.^6^ used logistic regression to establish a model for CTR prediction. McMahan et al.^7^ proposed a method to solve the Google’s ad problem and got better performance. Multiple features are used as input data such as ad information and keywords. Chapelle et al.^8^ used LR to solve the problem of prediction for Yahoo’s. The linear model is easy, but it can not capture feature interaction. To overcome the limitation, Factorization Machine^9^ (FM) and its variants^10^ are used to capture feature interactions and get better results. The field-aware factorization machines (FFM) introduced field aware latent vectors to capture feature interaction. However, the Factorization Machine model is relatively weak in obtaining high-order feature interaction. He et al.^11^ utilized decision trees and LR to improve the result. However, these models use shallow layer that have limited representation power of feature interactions.

Recently, deep neural networks have achieved great success in many research fields such as in computer vision^12,13^, image processing^14,15^ and natural language processing^16,17^. Therefore, researchers have proposed many CTR prediction models based on deep learning. How to effectively model feature interaction is an important problem in most models. Zhang et al.^18^ proposed the Factorization Machine based Neural Network (FNN). The model uses FM to pre-train the embedding layer based on forward neural network. FNN model has a better performance on capturing high-order feature interactions. Cheng et al.^19^ combines the linear and the deep neural network to capture feature interactions. The wide part of the model is still needed feature engineering. This means that feature interaction also needs to be designed manually. To solve the problem, DeepFM model^20^ uses FM to replace the wide part, and shared the same input. DeepFM model is considered to be the more advanced model in the field of CTR estimation. Product-based Neural Networks (PNN) model^21^ is used for user response prediction. The model utilizes a product layer and gets feature interaction. Lian et al.^22^ proposed a CIN model, and captured feature interactions at the vector-wise level. Deep and Cross Network (DCN)^23^ efficiently learns feature crossing and no manual feature engineering. Shan et al.^24^ found the relationship behind the user behavior based on the residual neural networks and proposed the deep crossing model^25^. In addition, some models also are proposed based on Convolutional Neural Networks (CNN). Kim et al.^26^ designed a multi-array CNN Model for ad CTR Prediction. This method can capture local feature information based on CNN. Wang et al.^27^ used CNN based on attention to find the different features. Ouyang^28^ considered each target ad independently and proposed MA-DNN model that achieved a better result.

In practical applications, different predictors usually have different predictive capabilities. Features that have a greater contribution to the prediction results should be given greater weights. As we all know, the attention mechanism^29^ has a powerful function in distinguishing importance of features. Wang et al.^30^ improves FM based on the attention mechanism to find the different importance of different features. Cao et al.^31^ proposed a Meta-Wrapper model that utilized the attention mechanism and capture the user interested items in historical behaviors. Xiao et al.^32^ builds Attentional Factorization Machine (AFM) model, the model can mine feature interaction based on neural attention network. However, the model ignored the important of user behavior for CTR prediction.

In summary, the high-order expression and interaction of features significantly improves the expression ability of features and the generalization ability of the models. However, in the process of capturing feature interactions, the influence of user interest is often ignored. Constructing a model to capture the user’s dynamics and evolving interests from the user’s sequential behavior has been widely proven effective in CTR prediction tasks. At the same time, Dynamic Quality of Service (QoS) prediction for services is currently a hot topic and a challenge for research in the fields of service recommendation and composition. Jin et al.^33^ addresses the problem with a Time-aWare service Quality Prediction method (named TWQP). Deep Interest Network (DIN)^34^ introduced the influence of user interests and found user interests based on user behaviors. DIN can capture the diversity characteristic of user interests and improve the performance of CTR prediction. In order to capture the dynamic evolution of user interests, Deep Interest Evolution Network (DIEN)^35^ was proposed. DIEN gets interest features and finds interest evolving process. Wang et al.^36^ presented a Trust-based Collaborative Filtering (TbCF) algorithm to perform basic rating prediction in a manner consistent with the existing CF methods. The algorithm employs multiple perspectives to extract proper services and achieves a good tradeoff between the robustness, accuracy, and diversity of the recommendation. Liu et al.^37^ proposed an attention-based bidirectional gated recurrent unit (GRU) model for point-of-interest (POI) category prediction (ABG_poic). They regard the user's POI category as the user's interest preference because the fuzzy POI category is easier to reflect the user interest than the POI. By modeling the user's sequential behavior, the feature representation is enriched, and the prediction accuracy is significantly improved.

The concept of session often appears in sequential recommendation, but it is rarely seen in CTR prediction tasks. Session-based recommendation achieves good results via user dynamic interest evolving. Neural Attentive Recommendation Machine (NARM)^38,39^ used an attention mechanism to capture the user purpose in the current session. Zhang et al.^40^ analyzes the current session information from multiple aspects and improves user satisfaction. However, most existing studies for CTR prediction ignore that the sequences are composed of sessions. Upon all these perspectives, we introduce a novel session interest model via self-attention (SISA) to get a better result for CTR.

## Material and methods

We propose Session Interest Model via Self-Attention (SISA) for CTR prediction, which uses multiple historical sessions to simulate the user’s sequential behavior in the CTR prediction task. The SISA model includes five modules, we describe session interest model via self-attention in this section. We first introduce feature representation and embedding in “Feature representation and embedding”. Next, “Session division module” illustrates the session division module. Then, we describe the session interest extractor module in “Session interest extractor module” and session interacting module in “Session interest interacting module”. Finally, “The overall architecture of SISA model” presents the structure of SISA model.

### Feature representation and embedding

We use four groups of features (User Profile, Scene Profile, Target Ad, User Behavior) as input data for the model. The encoding vector of the feature group can be expressed by $E \in {\mathbb{R}}^{{M \times d_{{{\text{model}}}} }}$, where $d_{{{\text{model}}}}$ is the embedding size and $M$ is the size of sparse features. Through feature embedding, User Profile can be represented by $X^{U} \in {\mathbb{R}}^{{N_{u} \times d_{{{\text{model}}}} }}$, where $N_{u}$ is the number of User Profile sparse features. Similarly, both Scene Profile and Target Ad can be expressed as $X^{{\text{S}}} \in {\mathbb{R}}^{{N_{s} \times d_{{{\text{model}}}} }}$,$X^{{\text{I}}} \in {\mathbb{R}}^{{N_{i} \times d_{{{\text{model}}}} }}$, where $N_{s}$ and $N_{i}$ are the number of Scene Profile and Target Ad sparse features, respectively. User Behavior is represented by $X{ = }[x_{1} ;...;x_{i} ;...x_{{\text{N}}} ] \in {\mathbb{R}}^{{N \times d_{{{\text{model}}}} }}$, where $N$ is the number of user historical behaviors and $x_{i}$ is the embedding of the $i$-th behavior.

### Session division module

In order to get the user session interests, we divide the user behavior sequences ${\text{X}}$ into sessions $S$, where the $k$-th session $S_{k} = [x_{1} ;...;x_{i} ;...x_{{\text{T}}} ] \in {\mathbb{R}}^{{T \times d_{{{\text{model}}}} }}$,$T$ is the number of behaviors in each session and ${\text{b}}_{i}$ is user $i$-th behaviors in current session. Many behaviors that are more than 30 min apart into user sessions follow by Grbovic’s^4^ method.

### Session interest extractor module

On the one hand, the behaviors in the same session are closely related to each other, on the other hand, the user random behavior in the session deviates from the original expression of session interest. In order to capture the inner relationship between behaviors in the same session and find the impact of those irrelevant behaviors, a multi-head self-attention mechanism^41^ is used for each session. At the same time, we apply positional encoding to the embedding based on self-attention mechanism.

So as to take advantage of order relationship of the sequence, self-attention mechanism is used to positional encoding to the input embedding. Also, we capture the sequence of sessions and the bias in different representation subspaces. So, we define bias encoding BE as follows:

1 $${\text{BE}}(u,k,t) = W_{u}^{U} + W_{k}^{K} + W_{t}^{T}$$

where $W^{U} \in {\mathbb{R}}^{{d_{{{\text{model}}}} }}$ is the bias vector of the unit position in the behavior embedding, and $u$ is the index of the unit in the behavior embedding. $W^{K} \in {\mathbb{R}}^{K}$ is the bias vector of session, $k$ is the index of session. $W^{T} \in {\mathbb{R}}^{T}$ is the bias vector of the position in the session. So user behavior sessions can be represented as follows via bias encoding:

2 $${\text{S = S}} + {\text{BE}}$$

As we all know, the user's click behavior is influenced by many factors, such as color, shape, and price. Multi-head self-attention can get the relationship in different representation subspaces. We use $S_{k} = [S_{k1} ;...;S_{kn} ;...S_{kN} ]$, where $S_{kn} \in {\mathbb{R}}^{{T \times d_{n} }}$ is the $n$-th head of $S_{k}$.$N$ is the number of heads, $d_{n} = \frac{1}{n}d_{{{\text{model}}}}$.Through these representations, we can calculate the output of $head_{n}$ as follows:

3 $$head_{n} = Attention(S_{kn} W^{Q} ,S_{kn} W^{K} ,S_{kn} W^{V} )$$

4 $$Attention(Q,K,V) = {{softmax}}\left(\frac{{S_{kn} W^{Q} W^{{K^{T} }} S_{kn}^{T} }}{{\sqrt {d^{{{{model}}}} } }}\right)S_{kn} W^{V}$$

where $W^{Q} ,W^{K} ,W^{V}$ are weight matrices. Then bias encoding-based feedforward neural network can further improve the nonlinear ability:

5 $$I_{k}^{S} = FNN(Concat(head_{1} ,...head_{N} )W^{O} )$$

6 $$I_{k} = Avg(I_{k}^{S} )$$

where $W^{O}$ is the weight matrix. $FNN( \cdot )$ is the feedforward neural network. ${\text{Avg}}( \cdot )$ is the average pooling. $I_{k}$ is the user $k$-th session interest.

### Session interest interacting module

The different session interest may be related to each other or follow a sequential pattern, GRU performs better at capturing sequential relationships, so we use GRU^42,43^ to capture the interaction and evolution of user different historical session interests.

The activation $h_{t}$ of the GRU at time $t$ is a linear interpolation between the previous activation $h_{{t{ - }1}}$ and the candidate activation $\tilde{h}_{t}$:

7 $$h_{t} = (1 - z_{t} )h_{t - 1} + z_{t} \tilde{h}_{t}$$

The update gate is represented by:

8 $$z_{t} = \sigma (W_{z} {\text{I}}_{t} + U_{z} h_{t - 1} + b_{z} )$$

Candidate activation $\tilde{h}_{t}$ is calculated as follows:

9 $$\tilde{h}_{t} = \tanh (WI_{t} + u(r_{t} \odot h_{t - 1} ) + b_{h} )$$

where $r_{t}$ is a set of reset gates and $\odot$ is an element-wise multiplication. When off ($r_{t}$ close to 0), the reset gate makes it forget the state of the previous calculation.

At the same time, the $r_{t}$ is represented as follows:

10 $$r_{t} = \sigma (W_{r} {\text{I}}_{t} + U_{r} h_{t - 1} + b_{r} )$$

where $\sigma$ is a sigmoid function, $I_{t}$ is the input of GRU, $W$ and $b$ are the parameters that are trained.

The hidden state $h_{t}$ can capture the dependency between session interests. However, the user's session interest related to the target ad has a greater impact on whether the user will click on the target ad. So the weight of the user's session interest needs to be reassigned to the target ad. We use attention mechanism for local activation and use GRU to model the representation of session interests and target ad.

We use $I_{t}^{\prime }$,$h_{t}^{\prime }$ represent the input and hidden state of GRU. The input of second GRU is corresponding state in the part of capturing session interest interaction: $I_{t}^{\prime } { = }h_{t}$. The attention function we used can be formulated as:

11 $$a_{k}^{I} = \frac{{\exp (I_{k} W^{I} X^{I} )}}{{\sum\nolimits_{k}^{K} {\exp (I_{k} W^{I} X^{I} )} }}$$

where $W^{I}$ has the corresponding shape, attention score can reflect the relationship between target ad ${\text{X}}^{I}$ and input $h_{t}$, and the more relevant ones will get more attention weight.

**Figure 1:**
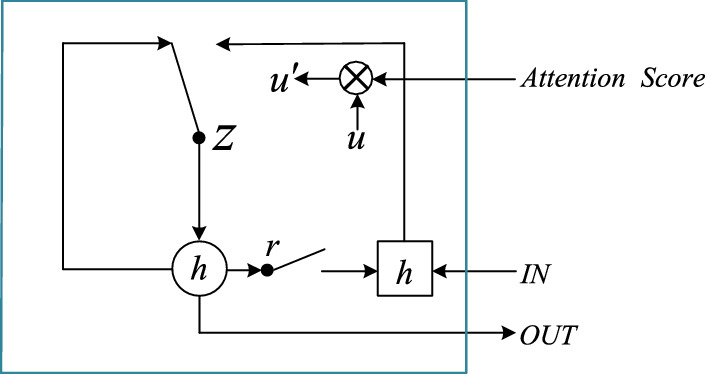
The architecture of AUGRU.

We combine attention mechanism and GRU and use the GRU with attentional update gate (AUGRU) to consider influences of between session interests and the target ad:

12 $$\tilde{u}_{t}^{\prime } = a_{k}^{I} * u^{\prime}_{t}$$

13 $$h_{t}^{\prime } = (1 - \tilde{u^{\prime}}_{t} ) \circ h^{\prime}_{t - 1} + \tilde{u^{\prime}}_{t} \circ \tilde{h^{\prime}}_{t}$$

where $u^{\prime}_{t}$ is the original update gate of AUGRU, $\tilde{u}_{t}^{\prime }$ is the attentional update gate that we use in AUGRU. $h_{t}^{\prime }$, $h^{\prime}_{t - 1}$ and $\tilde{h^{\prime}}_{t}$ are the hidden states of AUGRU. Figure 1 is the framework of GRU application attention mechanism (AUGRU).

By using AUGRU, we retain the original dimension information of the update gate. We measure all dimensions of the update gate by using attention score and consider the impact of different session interests on the target ad.

### The overall architecture of SISA model

**Figure 2:**
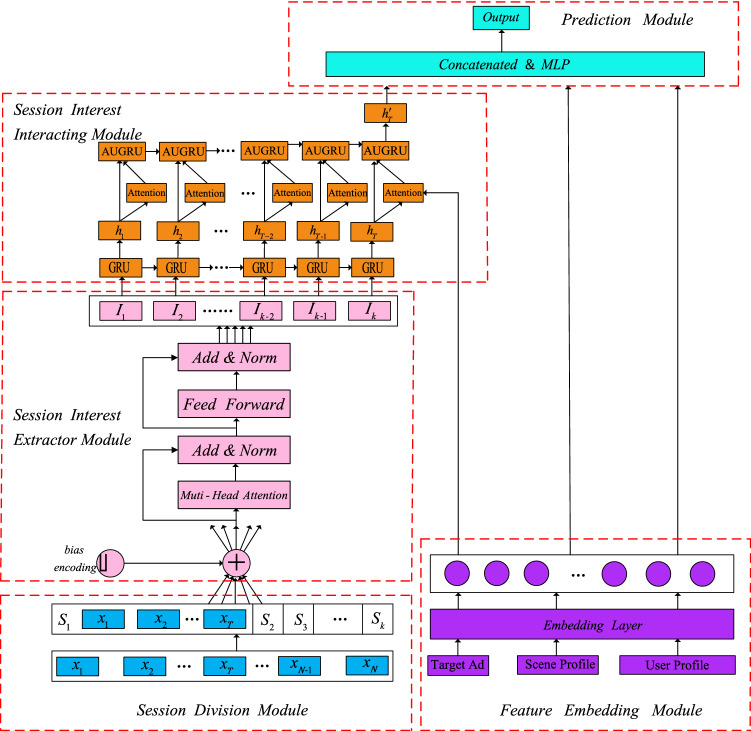
The structure of SISA.

The SISA model includes five modules, and the structure is shown in Fig. 2.

In feature representation and embedding module, informative features such as user profile, scene profile and target ad are transformed into dense vectors by using an embedding layer. In session division module, we divide user’s behavior sequences into sessions and get use’s session sequence. In session interest extractor module, we capture the inner relationship between behaviors in the same session. We employ multi-head self-attention to reduce the influence of unrelated behaviors. We also apply positional encoding to the input embedding based on self-attention mechanism and get order relations of the sequence. In session interest interacting module, we use GRU to capture the interaction and evolution of user different historical session interests. The user's session interest related to the target ad has a greater impact on whether the user will click on the target ad. So we use AUGRU to model the representation of session interests and target ad. In prediction module, embedding of sparse features and session interests that we capture are concatenated and then imported into MLP. Finally, the softmax function is used to get probability that people click on the ad.

The loss function is a negative log-likelihood function and is usually expressed as:

14 $$L = - \frac{1}{N}\sum\limits_{(x,y) \in D}^{N} {(y\log p(x)} + (1 - y)\log (1 - p(x)))$$

where $D$ denotes the training size $N$, $p(x)$ denotes the probability that the user clicks on an ad.

## Experiments

### Experiments setting

#### Datasets

In order to verify the effectiveness of SISA model, we conducted the experiments on two subsets of Amazon dataset^44^: Books and Electronics and two public datasets: Avazu and Criteo. In CTR model evaluation, most researchers often use Criteo dataset. The result of the different dataset is showed in Table 1. The datasets are randomly divided into three parts: training set (80%), validation set (10%) for adjusting hyperparameters, and test set (10%).

**Table 1:** Basic statistics of the datasets.

Dataset	Users	Items	Features	Samples
Books	53,126	46,783	48,632	297,659
Electronics	32,359	36,191	39,715	203,114
Avazu	80,724	71,473	127,694	854,261
Criteo	30,023	33,871	46,723	210,342

#### Evaluation metrics

In our experiment, we employ three metrics: AUC (Area Under ROC), Logloss and RMSE (Root Mean Square Error).

AUC: The area under the ROC curve is a more commonly used indicator for evaluating classification problems (such as CTR prediction)^45^. The value of AUC is larger, the result is better.

Logloss: Logloss is applied to calculate the distance in a binary classification problem. The value of logloss is smaller, the performance of the model is the better.

RMSE: RMSE^46^ can be defined as follows:

15 $$RMSE = \sqrt {\frac{1}{\left| T \right|}\sum\limits_{i} {(y_{i} - \hat{y}_{i} )}^{2} }$$

where $y_{i}^{t}$ is the observed scores and $\hat{y}_{i}^{t}$ is the value of prediction, *T* is the testing set. The score of RMSE is small, the result of the model is great.

#### Parameter settings

We employ dropout to prevent over-fitting in the neural networks and the value of dropout rate is 0.4. We set the size of the hidden state in the GRU is 56. At the same time, we use $10^{ - 4} ,10^{ - 3} ,10^{ - 2} ,10^{ - 1}$ as learning rates to test. Also, different number of neurons from 100 to 800 is employed.

### Comparisons with different models

**Figure 3:**
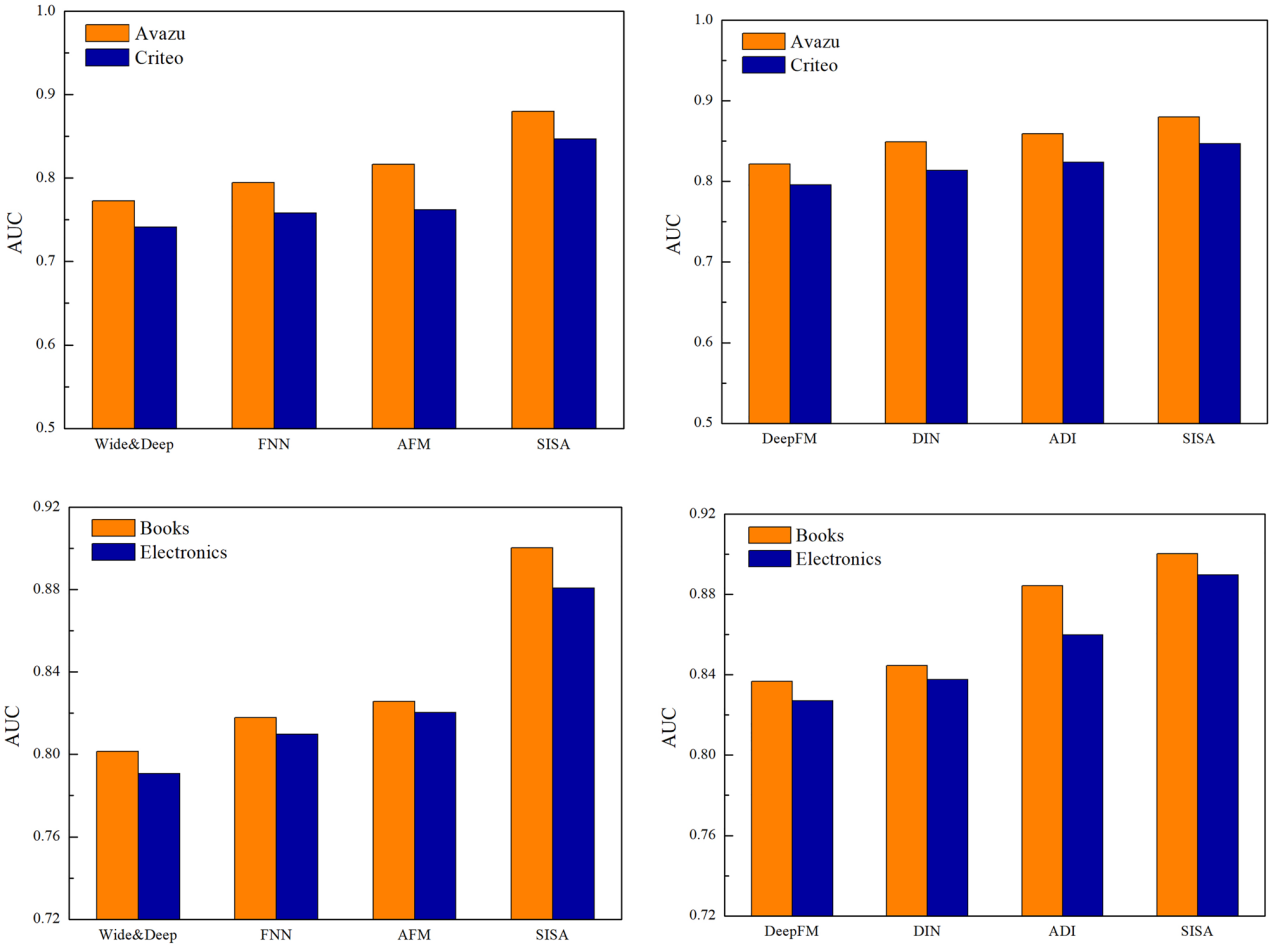
AUC performance comparison with other model.

To verify the efficiency of the SISA that we proposed, we compare SISA with some mainstream CTR prediction model. It shows the results of the different models for AUC in Fig. 3. Tables 2 and 3 show the value with logloss and RMSE, respectively. Through comparison, many aspects can be seen.

1. Wide&Deep^19^ is a model that has wide part and deep part. The linear part uses manually designed cross-product feature for better interactions. However, the wide part can not get the features automatically. So the Wide&Deep model does not have better performance in all the models.
2. FNN^18^ is a model that can get high-order features and uses deep learning to automatically learn feature interactions. Also, it improves the FM. However, when use the DNN, the model needs to train FM, so it has limitations.
3. AFM^32^ can find the importance of different feature interactions and use neural network to capture the feature interactions. As we all know, different feature interaction has different useful for results. The performance of AFM is better. This can be verified that using the attention mechanism can enhance performance of the model.
4. DeepFM^20^ is a new network framework that combines the FM and deep neural networks. It can model low-order feature interactions like FM and model high-order feature interactions like deep neural networks. DeepFM can be trained without any feature engineering. So the model outperforms both Wide&Deep and FNN.
5. DIN^34^ is a model for CTR prediction that exploits the rich historical behavior data to extract user interest. The model captures the feature interactions based on neural networks. It is based on the attention mechanism to get the representation of the user behavior and target ad. DIN has the better performance than DeepFM.
6. ADI^35^ captures interest evolving processes from user behaviors and gets higher prediction accuracy. However, the SISA model performs better than others. In SISA, we partition user behavior sequences into multiple sessions, user session interests follow a sequential pattern and more suitable for modeling. We can see that SISA model based on user session interest improves accuracy in all datasets. As we can see that in Avazu dataset the SISA increased by 1.8% compared to other models.

**Table 2:** Overall CTR prediction for Logloss performance in different datasets.

Model	Logloss			
	Books	Electronics	Avazu	Criteo
Wide&Deep	0.1213	0.1279	0.2237	0.3543
FNN	0.1201	0.1268	0.2204	0.3529
AFM	0.1197	0.1235	0.2189	0.3482
DeepFM	0.1134	0.1192	0.2145	0.3438
DIN	0.1123	0.1146	0.2107	0.3407
ADI	0.1014	0.1077	0.2083	0.3372
SISA	0.0978	0.1006	0.1964	0.3286

**Table 3:** Overall CTR prediction for RMSE performance in different datasets.

Model	RMSE			
	Books	Electronics	Avazu	Criteo
Wide&Deep	0.5071	0.5224	0.5326	0.6072
FNN	0.5043	0.5202	0.5302	0.5986
AFM	0.4996	0.5138	0.5279	0.5823
DeepFM	0.4942	0.5079	0.5213	0.5761
DIN	0.4578	0.4693	0.5185	0.5625
ADI	0.3675	0.4267	0.5061	0.5485
SISA	0.3121	0.3783	0.4925	0.5306

### Sensitivity analysis of the model parameters

We explore the influence of different parameters for the results of SISA model, such as the epoch, the number of neurons per layer, and the dropout rate $\beta$.

**Figure 4:**
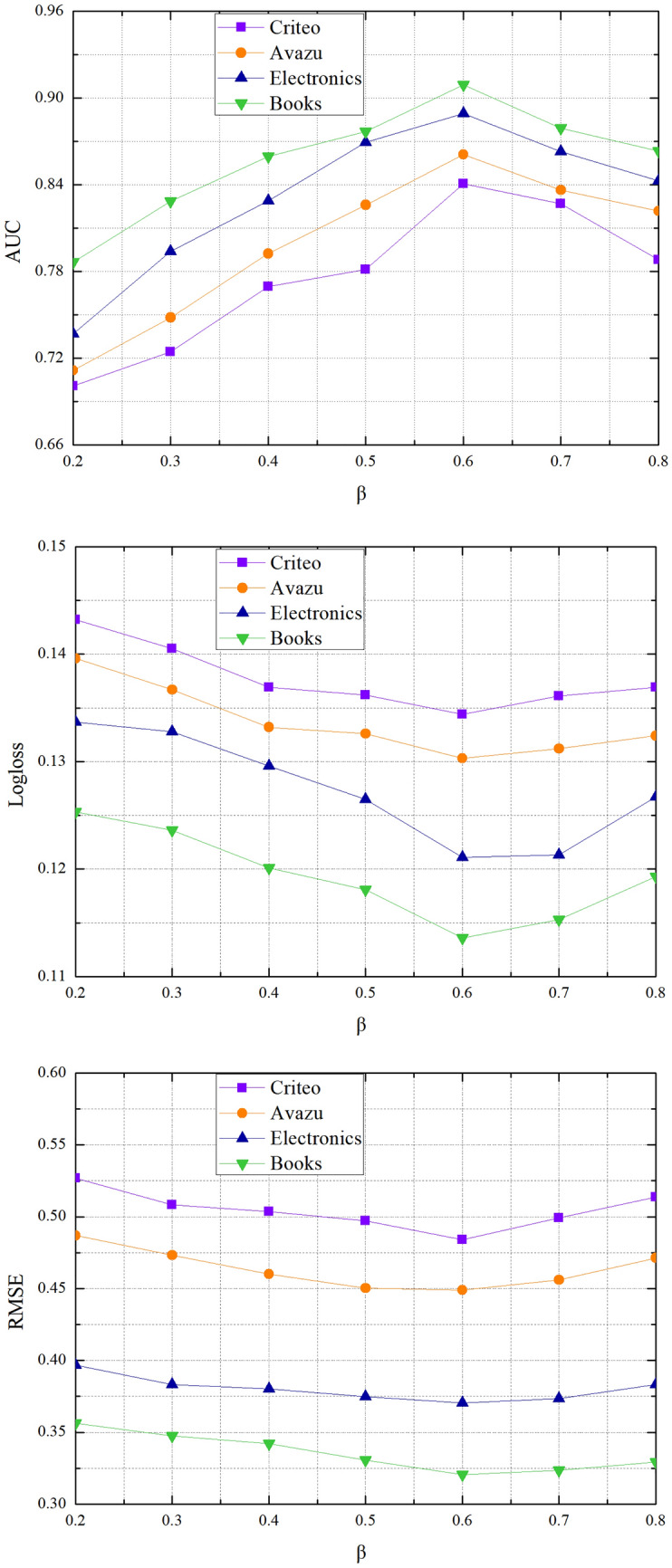
Performance comparisons w.r.t. the dropout rate $\beta$.

Dropout is the probability of neurons remaining in the network. First we set $\beta$ to be 0.2, 0.3, 0.4, 0.5, 0.6, 0.7, 0.8. As shown in Fig. 4, the $\beta$ can help SISA learn powerful features. The $\beta$ is properly set (from 0.5 to 0.8), the SISA model is able to reach its best performance at all datasets. However, with an increasing of the value of $\beta$, the performance of SISA shows a downward trend. So we choice $\beta { = }0.6$ in the following experiment.

**Figure 5:**
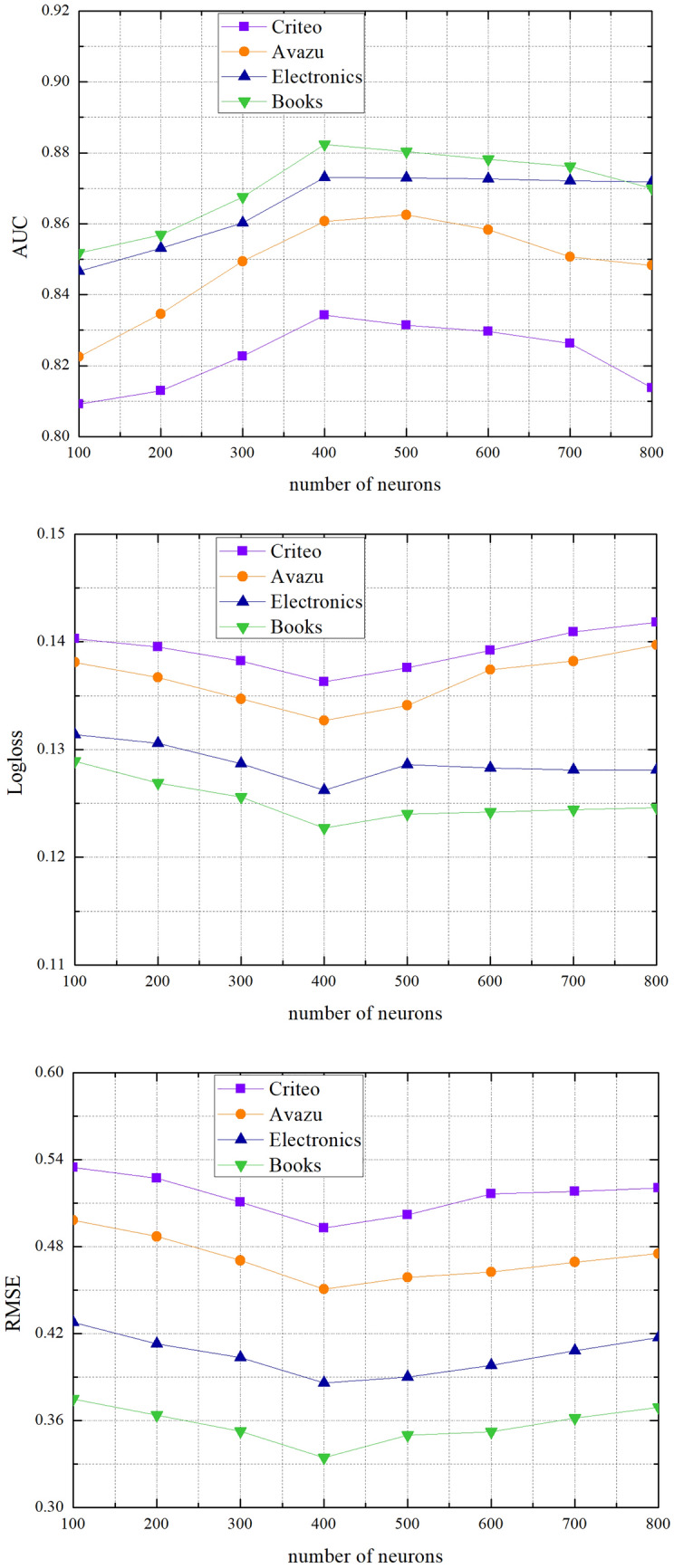
Performance comparisons w.r.t. the number of neurons.

When other factors remain the same, we study the effect of different number of neurons. In Fig. 5, it is not that the higher the number of neurons, the better the result. When the number of neurons is 500, 600 or 700, the performance of SISA is stably and even worse in all datasets. It is because that the model is overfit. So we select 400 in the experiment.

**Figure 6:**
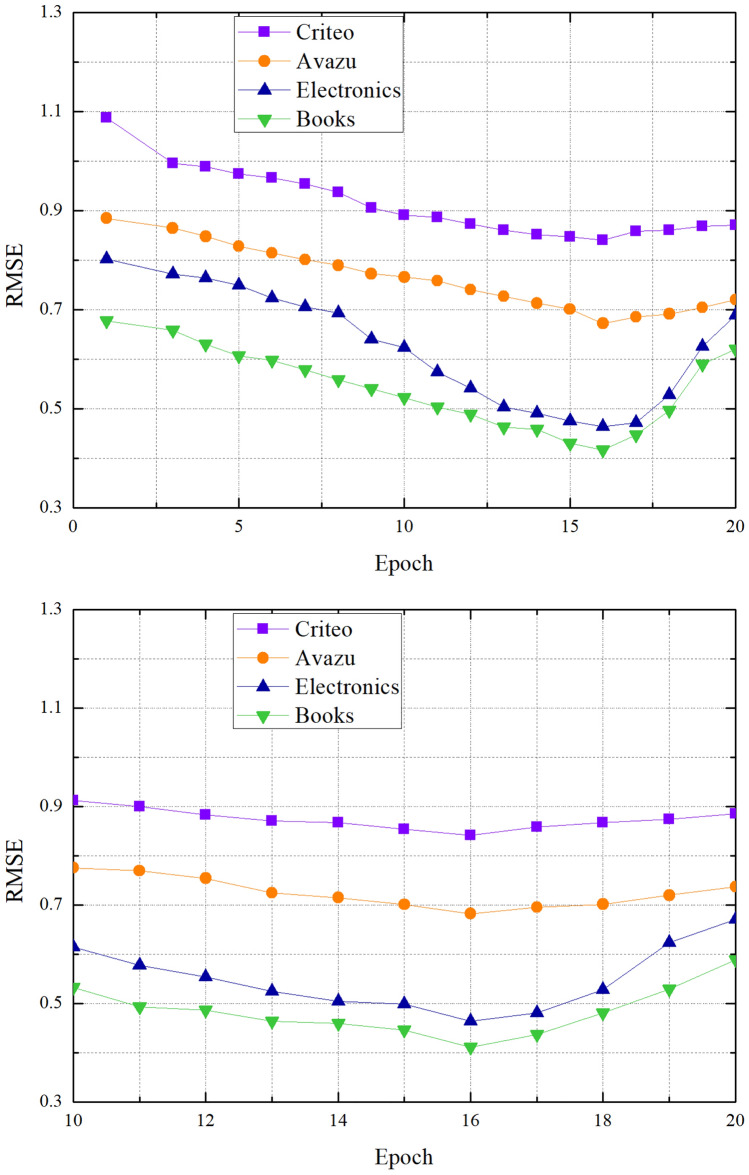
The effect of the epoch.

We study the influence of different epoch values for CTR prediction through experiments. Can see from Fig. 6, the model performed poorly when the value of epoch is 0–5. This is because the number of iterations is too small to determine the appropriate parameters. As the value of epoch increases, the value of RMSE becomes smaller. Compared with other datasets, the value of RMSE in the Amazon dataset fluctuates relatively high. Because model needs different numbers of features to train on different datasets, and the diversity of the data will cause some errors. If the value of epoch is not suitable, the performance of the model will fluctuate greatly. At the same time, the model has better performance when the value of epoch is between 10 and 20 in Fig. 6. Therefore, in the experiment we set the value of epoch to 16.

### Comparison among SISA variants

Although we have demonstrated strong empirical results, the results presented do not isolate the specific contributions of each component of SISA, so we conduct ablation experiments on SISA. In Table 4, SN stands for a network like the one used in FNN. IN stands for complex network that can get user session interest. AVG represents average pooling and MAX is maximum pooling strategies. The self-attention module uses AVG and MAX instead, and ATT-IN stands for the SISA model.

**Table 4:** AUC of SISA variants in different datasets.

Datasets	FNN	AVG		MAX		ATT	
		SN	IN	SN	IN	SN	IN
Avazu	0.7825	0.7841	0.7937	0.7902	0.8016	0.8083	0.8324
Criteo	0.7758	0.7702	0.7814	0.7843	0.7938	0.8173	0.8244
Books	0.8026	0.8272	0.8395	0.8331	0.8405	0.8501	0.8965
Electronics	0.7938	0.8198	0.8306	0.8274	0.8317	0.8457	0.8772

In Table 4, the IN-ATT model has the highest AUC value. FNN model uses neural networks to automatically capture feature interactions. The model of SN-AVG, -MAX, and -ATT ignore the impact of interest on click-through rate prediction, not as good as a model based on session interest. At the same time, some models do not distinguish the contribution of different features to the prediction results, so the performance of prediction is not good. SISA model has achieved better results by combining the user session interest with the self-attention mechanism.

## Conclusion

In this paper, we propose a new model, namely SISA, to model user session interest for CTR prediction. First, we specially divide the user sequential behavior into sessions and design session interest extractor module. We employ a self-attention network with bias coding to obtain an accurate expression of interest for each session. Then, we use GRU to get the interaction of different session interests. We exploit GRU with attentional update gate (AUGRU) to aggregate their target ad to find the influences of different session interests at session interacting module. Next, embedding of sparse features and session interests that we capture are concatenated and fed into MLP in prediction module. Experiment results prove the efficiency of SISA on different datasets. In the feature, we will pay attention to use knowledge graph to capture user interests and model click-through rate predictions.
